# Diversity of bacterial dimethylsulfoniopropionate degradation genes in surface seawater of Arctic Kongsfjorden

**DOI:** 10.1038/srep33031

**Published:** 2016-09-08

**Authors:** Yin-Xin Zeng, Zong-Yun Qiao, Yong Yu, Hui-Rong Li, Wei Luo

**Affiliations:** 1 Key Laboratory for Polar Science of State Oceanic Administration, Polar Research Institute of China, Shanghai 200136, China

## Abstract

Dimethylsulfoniopropionate (DMSP), which is the major source of organic sulfur in the world’s oceans, plays a significant role in the global sulfur cycle. This compound is rapidly degraded by marine bacteria either by cleavage to dimethylsulfide (DMS) or demethylation to 3-methylmercaptopropionate (MMPA). The diversity of genes encoding bacterial demethylation (*dmdA*) and DMS production (*dddL* and *dddP*) were measured in Arctic Kongsfjorden. Both *dmdA* and *dddL* genes were detected in all stations along a transect from the outer to the inner fjord, while *dddP* gene was only found in the outer and middle parts of the fjord. The *dmdA* gene was completely confined to the *Roseobacter* clade, while the *dddL* gene was confined to the genus *Sulfitobacter*. Although the *dddP* gene pool was also dominated by homologs from the *Roseobacter* clade, there were a few *dddP* genes showing close relationships to both Alphaproteobacter and Gammaproteobacter. The results of this study suggest that the *Roseobacter* clade may play an important role in DMSP catabolism via both demethylation and cleavage pathways in surface waters of Kongsfjorden during summer.

Dimethylsulfoniopropionate (DMSP) is an organic sulfur compound that occurs worldwide in large amounts (10^9^ tons or more per year) in marine algae and plants^1^, where it may function as an osmolyte, cryoprotectant, predator deterrent, and antioxidant^2^,^3^,^4^,^5^. When released into seawater, DMSP is rapidly catabolised by bacteria via two enzymatic pathways: demethylation and cleavage^6^,^7^,^8^. The demethylation pathway converts a large fraction (50 to 90%) of dissolved DMSP to 3-methylmercaptopropionate (MMPA), which is subsequently incorporated into the biomass of microbial cells^7^,^9^. Over half of the bacterioplankton cells in ocean surface waters are capable of conducting demethylation of the phytoplankton metabolite DMSP^10^. The alternative cleavage pathway transforms the remaining part of the dissolved DMSP via various DMSP lyases to dimethylsulfide (DMS) and acrylate (or 3-hydroxypropionate)^11^,^12^. DMS represents the most important natural source of sulfur to the atmosphere, where its oxidation products cause cloud nucleation and may affect weather and climate^13^. DMS emissions can exert a greater influence on climate in regions in which low aerosol concentrations prevail, such as in the Arctic during summer^14^.

The only gene found to encode the first step in the DMSP demethylation pathway (*dmdA*) is known to be harboured by the *Roseobacter* clade, SAR11, SAR116 member “*Candidatus* Puniceispirillum marinum” IMCC1322, and Gammproteobacteria^10^,^15^,^16^. To date, seven DMSP lyase genes involved in the DMSP cleavage pathway, *dddD*, *dddK*, *dddL*, *dddP*, *dddQ*, *dddY*, and *dddW*, have been identified from bacterial isolates^1^,^17^. Among the DMSP lyase genes, *dddP* and *dddQ* are the most abundant *ddd* genes in the Global Ocean Sampling (GOS) data set^18^,^19^. The *dddP* gene occurs frequently in marine Alphaproteobacteria (e.g., the *Roseobacter* clade, SAR11 and SAR116), as well as in Gammaproteobacteria and some Ascomycete fungi^1^,^17^,^18^,^20^,^21^. The *dddQ* gene is found in the *Roseobacter* clade and SAR11^1^,^17^. To date, most of our understanding of microbial DMSP metabolism in marine environments has come from studies conducted at low and mid-latitudes. Recent studies in Arctic waters indicate that bacteria primarily use DMSP as a carbon source, resulting in conversion efficiencies of DMSP into DMS of up to 31%^22^,^23^. However, studies of the abundance, diversity and biogeography of DMSP degradation genes in high-latitude marine environments remain insufficient.

Kongsfjorden is a glacial fjord in the Arctic situated on the western side of Spitsbergen in the Svalbard archipelago that is influenced by the inflow of transformed Atlantic water as well as by meltwater of glacial origin (Fig. 1)^24^. In early summer in a post-spring bloom environment, DMSP, DMS, and chlorophyll *a* concentrations in water are 10 nmol l^−1^, 1.5 nmol l^−1^ and 0.21 μg l^−1^, respectively^25^. To describe the distribution of DMSP degradation genes relative to surface water and identify the bacterial communities potentially associated with these genes, we collected seawater samples following transect from the outer to the inner part of Kongsfjorden and studied the diversity of *dmdA*, *dddL* and *dddP* genes using a clone library method.

## Results

### Microbial cell abundance

Based on DAPI analysis, the microbial cell abundance in Kongsfjorden ranged from 1.40 × 10^9^ to 3.26 × 10^9 ^cells l^−1^ and was highest at inner fjord station K5 (Table 1). To investigate the abundance of eubacteria and archaebacteria in surface seawater, sample K3 was analyzed using oligoFISH probes EUB338 and ARCH915. Remarkably, eubacterial abundance (on average 1.20 × 10^9^ cells l^−1^) was one order of magnitude higher than archaebacterial abundance (on average 1.43 × 10^8^ cells l^−1^). High detection of DAPI-stained cells by EUB338 using oligoFISH has been reported from surface samples collected from the California coast^26^. The percentage of bacteria detected by probe EUB338 was 85% of the cells detected with DAPI for sample K3. OligoFISH probe ARCH915 can unspecifically stain a population of marine bacteria^27^. The results of this study are consistent with those of a previous study of the prokaryotic community in surface waters that showed the population in Kongsfjorden was dominated by bacteria, while archaea only accounted for 1% of the DAPI-stained cells^28^.

### Primer test for *dmdA* gene amplification

To check the validity of primers for *dmdA* gene amplification, primer pairs dmdAUF160 and dmdAUR697, and dmdA282F and dmdA591R were used on reference strain *Ruegeria pomeroyi* DSS-3, which contains the identified *dmdA* gene^29^. Using primer pair dmdAUF160 and dmdAUR697, two PCR product bands (580 bp and 373 bp) were obtained with significant similarity (99.7–100%) to the 23S rRNA gene and trimethylamine methyltransferase gene of *Ruegeria pomeroyi* DSS-3. Non-*dmdA* sequences captured by the universal *dmdA* primer set dmdAUF160 and dmdAUR697 have also been found in Gulf of Mexico seawater microcosoms^10^. In contrast, analysis with primer pair dmdA282F and dmdA591R produced a single band of 309 bp with 99.6% sequence similarity to the *dmdA* gene of *Ruegeria pomeroyi* DSS-3. Therefore, primers dmdA282F and dmdA591R were used in this study for *dmdA* gene clone library construction.

### Bacterial *dmdA* gene clone libraries and distribution of *dmdA* genes

Three *dmdA* gene clone libraries were constructed from the environmental DNA of surface water samples along a transect from the outer (K1) to the inner (K5) part of the fjord (Table 2). A total of 263 clones showing a single band of approximately 306 bp resulted in107 operational taxonomic units (OTUs). Among them, three OTUs shared in the three libraries, accounted for 17%, 18% and 14% of clone library K1, K3 and K5, respectively. Shannon diversity index was higher at outer fjord station K1. However, pairwise comparison revealed no significant differences among the three libraries.

Based on 90% amino acid identity, a phylogenetic tree of these DmdA clone sequences (Fig. 2) yielded 19 major clusters (termed A1 to A8, B1 to B8, and C1 to C3), accounting for 95% of the total clone sequences. All sequences were distributed within the putative *dmdA* genes of strains belonging to the *Roseobacter* clade within the *Rhodobacteraceae* family. Clones affiliated with the genera *Sulfitobacter* (including Clusters A1, A2, A3, A4, B1, B2, B3 and C1) and *Roseovarius* (i.e., Cluster C2), and a member of the *Roseobacter* clade (i.e., Cluster C3) accounted for 28%, 25%, and 18% of the total clones, respectively. In addition, *dmdA* genotypes within Clusters C1, C2 and C3 found in all samples accounted for more than 44% of the total clones. Sequences affiliated with the genera *Rhodobacter* and *Thalassobius* were only observed in sample K1. However, sequences within Cluster B5 were more frequently observed at the inner station K5 than at the outer station K1.

### Bacterial *dddL* gene clone libraries and distribution of *dddL* genes

A total of 200 clones showing a single band of 143–339 bp were sequenced. Most of these were annotated as hypothetical protein, methionine-tRNA ligase and other enzymes, while only a total of ten clones showing a single band of 244 bp were distributed within the putative *dddL* genes of the genus *Sulfitobacter* (Fig. 3). The *dddL* gene sequences in genus *Sulfitobacter* were found in all investigated stations.

### Bacterial *dddP* gene clone libraries and distribution of *dddP* genes

Two *dddP* gene clone libraries were constructed from samples from the outer (K1) and middle (K3) stations. No PCR product or a concentration of product too low was observed in sample K5, resulting in a failure of *dddP* gene clone library construction. A total of 87 clones showing a single band of approximately 835 bp resulted in 37 OTUs. Among them, three OTUs shared by the two libraries accounted for 52% and 69% of clone libraries K1 and K3, respectively. Pairwise comparison showed that there was no significant difference between the two libraries.

Based on 81% amino acid identity, a phylogenetic tree of these DddP clone sequences (Fig. 4) yielded three clusters (termed P1 to P3), which were shared by the two libraries. All sequences were distributed within the putative *dddP* genes. Sequences within Cluster P1 accounted for 96% and 84% of clone libraries K1 and K3, respectively, and showed high sequence similarities to DddP of *Roseovarius nubinhibens* (NCBI: WP_009813101). Genotypes within Clusters P2 and P3 were also affiliated with the *Roseobacter* clade. The DddP sequences formed a cluster separately from the cluster of DddD sequences from *Pseudomonas* species (Fig. 4).

## Discussion

In Kongsfjorden, maximum bacterial abundance occurs in May and July^30^. Consistent with a previous study that showed the prokaryotic community in Kongsfjorden was dominated by bacteria^28^, the results of this study showed that bacteria was present in levels at least one order of magnitude more abundant than archaea, indicating that they may play a more important ecological role in the planktonic microbial community in Kongsfjorden. A 16S rRNA gene-based pyrosequencing survey revealed that sequences affiliated with the *Roseobacter* clade, including *Loktanella*, *Octadecabacter*, *Paracoccus*, *Phaeobacter*, *Roseobacter* and *Sulfitobacter*, comprise only a small percentage (2.3–6.9%) of the total clones at each station in the fjord^31^. However, *Roseobacter* clade members accounted for 97.7% of the Alphaproteobacteria^31^. In the present study, *dmdA* genotypes, and the identified *dddL* genotype were all clustered into the *Roseobacter* clade. In addition, sequences affiliated with the *Roseobacter* clade dominated *dddP* genotypes in the fjord. Previous study has shown that the *Roseobacter* groups play a dominant role in DMS production via DddP lyases in coastal waters of the northwestern Pacific Ocean^20^. These findings suggest that, despite its low abundance, the *Roseobacter* clade plays an important role in degradation of DMSP in surface waters of Kongsfjorden.

The DMSP demethylase gene (*dmdA*) is highly abundant in surface ocean waters^15^,^29^, while the DMSP lyase genes, including *dddD*, *dddL* and *dddP*, are in low abundance in bacteria from surface ocean waters^15^,^18^. When compared to *dmdA* sequences, which were detected at all investigated stations in Kongsfjorden, no *dddP* sequences were found at the inner station, and only a few identified *dddL* sequences were detected in this study. Previous studies have shown that the major route of bacterially-mediated DMSP catabolism in marine environments is via a demethylation process^7^,^32^,^33^, suggesting that most of the DMSP sulfur may also be assimilated by bacteria in Kongsfjorden. However, further study is required to perform quantitative analysis of the DMSP degradation genes to ascertain which catabolism pathway contributes more to the DMSP degradation in Kongsfjorden. A study using a ^35^S DMSP tracer to track the partitioning of DMSP sulfur to various products in oceanic and coastal seawater demonstrated that 60% of DMSP was assimilated by cells in coastal samples, while only a small portion of the DMSP was routed through the DMSP-cleavage pathway and DMS production^34^. Similar results have reported that DMS production from DMSP is a minor bacterial metabolic pathway in the Canadian High Arctic from northern Baffin Bay to the Beaufort Sea, as well as in Lancaster Sound^22^,^23^. In contrast, only 16% of DMSP was assimilated in ocean samples, while the remainder was transformed to dissolved non-volatile products^34^, which were probably formed by oxidation to DMSO and sulfate^35^. Cells in the coastal areas are likely to have higher growth rates and therefore increased sulfur demand, causing more sulfur to be incorporated into protein^35^. The primary sources of DMSP in marine surface waters are micro and macro-algae^36^. In Kongsfjorden, the maximum abundance of plankton occurs in July^30^. The capacity of the microbial community to take up dissolved DMSP and convert it into DMS increased with increasing phytoplankton biomass^22^. In Kongsfjorden, elevated DMSP production at high hydrogen concentration was largely a consequence of increased dinoflagellate biomass, and in particular, the increased abundance of the species *Heterocapsa rotundata*^25^. However, the increased DMSP production was not transformed into higher DMS concentrations. Because bacteria can both release DMS from DMSP and consume DMS as a major sink for DMS in the ocean^37^,^38^, this may have been a function of both increased demand for DMSP driven by elevated bacterial production and increased bacterial DMS consumption^25^. Bacteria consume DMSP in proportion to their abundance^23^. DMSP consumed by bacteria can then be routed through either one of two competing pathways, the demethylation or cleavage pathways. Experiments conducted with radiolabeled ^35^S-DMSP showed that bacteria switch from the demethylation pathway to the production of DMS when their sulfur demand is satisfied^39^.

The *dmdA* gene is largely confined to two groups of marine bacteria, the Roseobacters and the SAR11 clade^1^,^35^. Like the Roseobacters (e.g. *Ruegeria pomeroyi* DSS-3), *Pelagibacterales* strains of SAR11 have both the DMSP demethylation and cleavage pathways^17^. However, in Kongsfjorden, the *dmdA* genes in surface seawaters were entirely confined to the Roseobacters, showing a difference from the tropical and subtropical Pacific Ocean, where SAR11 bacterioplankton dominate the *dmdA* gene pool^40^,^41^. No sequence affiliated with SAR11 was detected in Kongsfjorden by pyrosequencing of 16S rRNA genes, whilst the Roseobacters dominated Alphaproteobacteria in bacterioplankton community^31^. Sequences affiliated with the genera *Sulfitobacter* and *Roseovarius* dominated the *dmdA* genotypes in Kongfjorden. At the same time, *dddL* sequences were confined to the genus *Sulfitobacter*, while sequences in the genus *Roseovarius* dominated *dddP* genotypes. Previous study of bacterioplankton communities in Kongsfjorden shows that sequences affiliated with the genus *Sulfitobacter* accounted for 55.8% of the Alphaproteobacteria^31^. However, sequence affiliated with the genus *Roseovarius* was not detected in bacterioplankton community^31^. Members of the *Roseobacter* clade, including the genera *Roseobacter*, *Roseovarius* and *Ruegeria*, have been found to can both demethylate and cleave DMSP^11^,^18^,^42^. Further study is required to examine whether both demethylation and cleavage pathways for DMSP occur in the same bacterium of the genus *Sulfitobacter*.

The *dddP* gene is one of the most frequently detected *ddd* genes in marine bacteria and is mainly found in the *Roseobacter* and SAR116 clades of Alphaproteobacteria^18^,^20^,^43^. However, evidence for horizontal gene transfer of *dddP* to some Gammaproteobacteria^21^ and fungal species^18^ has been reported. In the present study, the DddP sequence of *Pseudomonas* sp. DMSP-1, which is able to cleave DMSP into DMS and acrylate (data not shown), was found to be closely related to Cluster P2 and *Roseicitreum antarcticum* ZS2-28 (Fig. 4), suggesting a possible inter-class horizontal gene transfer of *dddP* between Alpha and Gammaproteobacteria. In addition, comparing with the absence of the genus *Roseovarius*, which dominated *dddP* genotypes in this study, the genera *Sulfitobacter* and *Loktanella* were the dominant members of the Alphaproteobacteria in bacterioplankton community in Kongsfjorden^31^, suggesting a possible inter-genus horizontal gene transfer of *dddP* in Alphaproteobacteria. Further study is required to verify the horizontal gene transfer of *dddP* to some bacteria in the seawater of Kongsfjorden.

Previous study shows that cloned sequences amplified with primer pair dddP_277F and dddP_1112R are distributed within Alphaproteobacteria and fungi^43^. Our study on Antarctic samples (unpublished) also showed that cloned *dddP* sequences were distributed within Alphaproteobacteria and Gammproteobacteria. In addition, amplified with primer set dddLf and dddLr, *dddL* genes were detected in *Pseudomonas* within the Gammaproteobacteria^44^ and *Sulfitobacter* within the Alphaproteobacteria in this study. Those results indicate that the primer sets used for amplification of *dddL* and *dddP* genes are not too biased towards the *Roseobacter* clade gene forms. Amplified with a universal *dmdA* primer set dmdAUF160 and dmdAUR697, a total of 239 sequences affiliated with *Ruegeria pomeroyi* DSS-3 were found in Gulf of Mexico^10^. However, in this study *dmdA* gene was not detected in *R. pomeroyi* DSS-3 using primer set dmdAUF160 and dmdAUR697. Though *dmdA* gene was successfully detected in *R. pomeroyi* DSS-3 using primer set dmdA282F and dmdA591R in this study, further study is required to check the specificity and coverage of the newly designed primers in the future, because that primer set might only capture *dmdA* genes of the marine *Roseobacter* clade.

The diversity of *dmdA*, *dddL* and *dddP* genes in surface seawaters of Kongsfjorden showed that planktonic bacteria in this Arctic fjord possess DMSP degradation genes for both demethylation and cleavage pathways. The *Roseobacter* clade (represented by the genera *Sulfitobacter* and *Roseovarius*) may play an important role in bacterial DMSP consumption. As PCR amplification is not quantitative in the same way as metagenomic data sets, a catalyzed reporter deposition-fluorescence *in situ* hybridization (CARD-FISH) approach should be considered for quantification of planktonic marine bacteria harbouring different DMSP degradation genes in further studies. In the marine environment, the prevalence of different bacterial DMSP degradation pathways is regulated by a complex set of factors including UV-A dose, temperature, carbon supply, and DMSP availability in relation to bacterial sulfur demand^6^,^45^,^46^. However, regulation mechanisms of the competing DMSP degradation pathways have not yet been thoroughly understood. A previous study shows that the best correlate with shifts of the dominant *dmdA* clades in response to an induced phytoplankton bloom was chlorophyll *a* concentration^11^. Thus, further studies of the abundance and distribution of DMSP degradation genes and the corresponding bacterial community structure in Kongsfjorde before, during, and after the phytoplankton bloom would help our understanding of DMSP catabolism pathways and environmental factors regulating these pathways in Arctic coastal waters.

## Methods

### Sample collection

**Figure 1:**
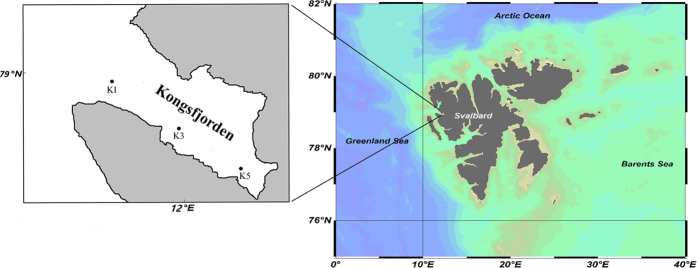
Maps of sampling sites in Kongsfjorden, Svalbard. The left image was created using CorelDRAW 8 software, and the right image was created using Ocean Data View 4.1.3 (https://odv.awi.de/).

**Figure 2:**
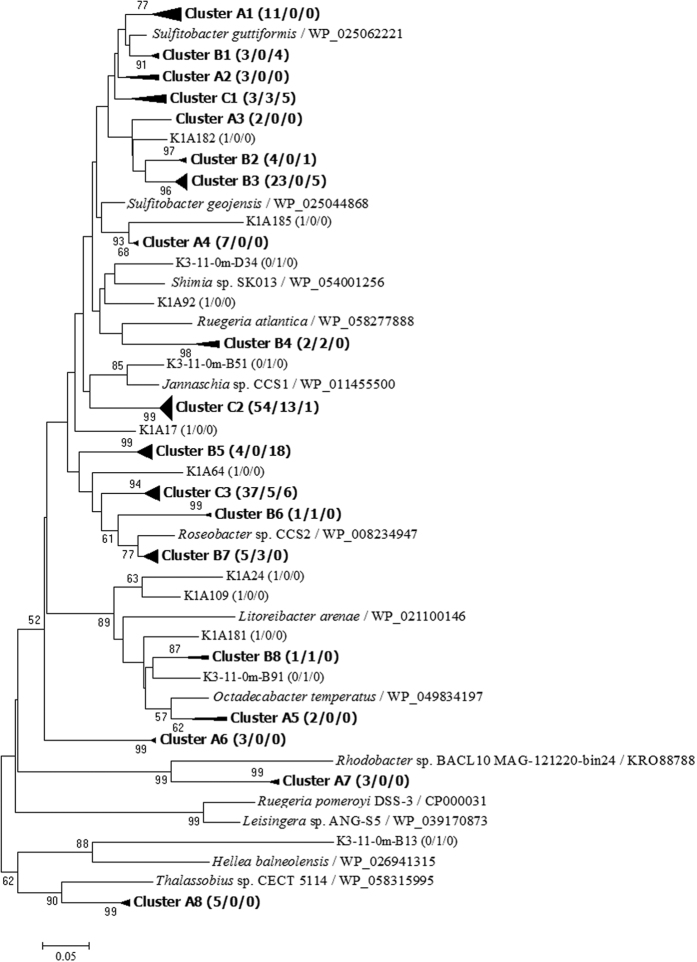
Phylogenetic tree of deduced DmdA sequences from three clone libraries of seawater in Kongsfjorden as well as those from known bacterial species available in NCBI. Cluster A indicates genotypes only observed in station K1, Cluster B indicates genotypes shared by stations K1 and K3 (or K5), and Cluster C genotypes shared among all stations. Bootstrap values of <50 have been removed for clarity. Numbers in parentheses following clone names or cluster names indicate the number of sequences found in stations K1, K3, and K5, respectively. The scale bar indicates evolutionary distance.

**Figure 3:**
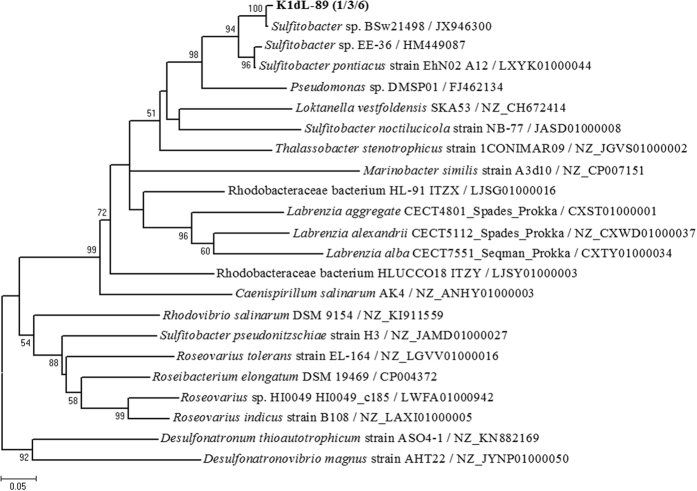
Phylogenetic tree of *dddL* gene sequences from three clone libraries of seawater in Kongsfjorden as well as those from known bacterial species available in NCBI. Bootstrap values of <50 have been removed for clarity. Numbers in parentheses following clone name indicate the number of sequences found in stations K1, K3, and K5, respectively. The scale bar indicates evolutionary distance.

**Figure 4:**
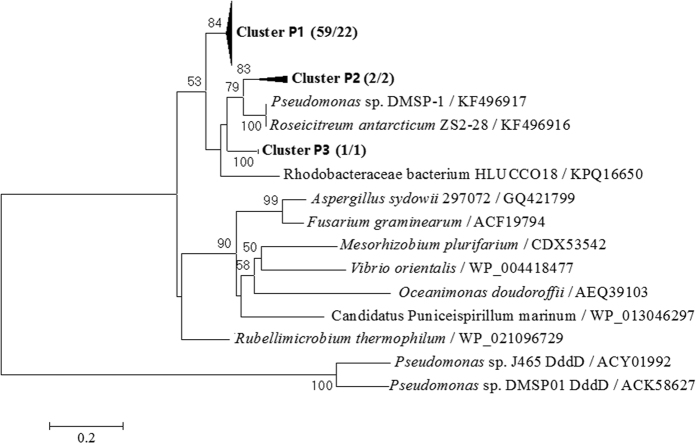
Phylogenetic tree of deduced DddP sequences from two clone libraries of seawater in Kongsfjorden plus those from known bacterial species available in NCBI. DddD sequences from *Pseudomonas* species were used as an outgroup. Bootstrap values of <50 have been removed for clarity. Numbers in parentheses following cluster names indicate the number of sequences found in stations K1 and K3, respectively. The scale bar indicates evolutionary distance.

Surface water samples were collected along a transect from the outer (K1) to the inner part (K5) of Kongsfjorden during the Chinese Arctic Yellow River Station Expedition in July of 2011 (Fig. 1). Sampling locations, physical factors and biogeochemical properties of the samples are summarized in Table 1. After collection, 50 ml of each water sample were fixed with buffered glutaraldehyde to a final concentration of 2% for 18 h at 4 °C, then filtered onto 0.2 μm pore-sized black polycarbonate membrane (Millipore, Germany) under low vacuum (<150 mm Hg). In addition, a 50 ml water subsample of K3 fixed with buffered glutaraldehyde was filtered onto 0.2 μm pore-sized white polycarbonate membrane (Millipore, Germany). Filters were air dried and stored at −20 °C until further processing. Microorganisms present in the sample were collected by filtration of 500 ml water onto 0.2 μm pore-sized Nucleopore filters (Whatman, UK). The filters were placed in sterile 2 ml centrifuge tubes and covered with 1.5 ml lysis buffer^47^. After processing, the tubes were immediately frozen and stored at −80 °C until DNA extraction.

### Microscopic microbial enumeration

Prokaryote abundance was determined by direct microscopic counts using the 4′,6-diamidino-2-phenylindole (DAPI) staining technique^48^. Black polycarbonate membrane filters were stained with the fluorochrome DAPI for 5 min at a concentration of 10 μg ml^−1^ in the dark. Two carbonindocyanine 3 (CY3)-labelled oligonucleotide probes, EUB338 and ARCH915, which are targeted to bacteria and to archaea, respectively^49^,^50^, were used for fluorescence *in situ* hybridization (FISH) counts of environmental sample K3. Sections of white polycarbonate membrane filters were hybridized as previously described^51^ using the CY3-labeled probes EUB338 and ARCH915. Microscopic counting was conducted using an epifluorescence microscope (Nikon ECLIPSE 80i, Japan) fitted with a digital camera (Nikon DXM1200F, Japan) under green light (detection of CY3-labelled cells) and UV light (DAPI-stained cells).

### DNA extraction

Total community DNA extraction was conducted as described by Bosshard *et al.*^47^. The purity and concentration of the extracted DNA were evaluated by use of a Nanodrop 2000 spectrophotometer (Thermo Scientific, Denmark). The molecular weight of DNA in extracts was determined by electrophoresing portions of extracts on 1.5% agarose gels using a 1 kb ladder (Promega, USA) as a size marker.

### Amplification of *dmdA* and *ddd* genes

Two universal primer pairs, dmdAUF160 and dmdAUR697^16^, and dmdA282F (5′-TGCTSTSAACGAYCCSGT-3′) and dmdA591R (5′-ACRTAGAYYTCRAAVCCBCCYT-3′), were used for *dmdA* gene amplification. Additionally, the universal primer pair dddLf and dddLr^44^ was used for *dddL* gene amplification and the universal primer pair dddP_277F and dddP_1112R^43^ was used for *dddP* gene amplification. Genomic DNA from the reference strain *Ruegeria pomeroyi* DSS-3, which possesses a *dmdA* gene of 1095 bp and a *dddP* gene of 1182 bp, was used as a template for standards. Both distilled water and genomic DNA from *Escherichia coli* DH5α were used as a template for negative control.

Amplification was conducted in 25 μl reactions with a Mastercycle Gradient Instrument (Eppendorf, Germany). All PCRs were performed in duplicate using 10–100 ng template DNA, and products were pooled before purification. *dmdA* gene amplification was conducted using primer pair dmdAUF160 and dmdAUR697 as described by Varaljay *et al.*^16^. PCR using the dmdA282F and dmdA591R primer set was conducted using a reaction mixture composed of 4.0 μl of template DNA, 2.5μl of 10 × PCR buffer (Mg^2+^ plus; TaKaRa, China), 100 μM deoxynucleoside triphosphates (dNTPs), 5 μg bovine serum albumin (BSA), 1 U of *Taq* DNA polymerase (TaKaRa, China), and 0.4 μM of each described primer. PCR conditions were as follows: initial denaturation at 94 °C for 3 min, followed by 35 cycles of denaturing at 94 °C for 1 min, annealing at 54 °C for 30 s, extension at 72 °C for 45 s, and final extension at 72 °C for 10 min.

*dddL* gene amplification was performed as described by Raina *et al.*^44^. The PCR reagent concentrations were identical to those described for *dmdA* gene amplification using primer pair dmdA282F and dmdA591R except that BSA was absent. The PCR reaction mixture for the *dddP* gene consisted of 4.0 μl of template DNA, 12.5 μl of DreamTaq Green PCR Master mix (2×; Thermo Scientific, Germany), 5 μg BSA, and 0.4 μM of each described primer. PCR conditions were identical to those described above except that annealing was conducted at 55 °C for 30 s and extension at 72 °C for 1 min. PCR products were visualized by electrophoresis on a 1% agarose gel stained with ethidium bromide.

### Clone library construction and sequencing

PCR products from seawater samples were purified using a gel purification kit (TaKaRa, China). Purified DNA was ligated into a pMD18-T cloning vector (TaKaRa, China) according to the manufacturer’s instructions, then used to transform *Escherichia coli* DH5α competent host cells. Only plasmid clones containing a target insert were sent to Majorbio Bio-pharm Technology Co., Ltd (Shanghai, China) for sequencing. Clones with inserts of the wrong size or chimeric sequences were removed from subsequent analyses.

### Data analysis

As the known DddPs with a similarity >81% from different bacteria belong to the same genus^43^, sequences showing more than 81% amino acid identity with each other were grouped in the same cluster. For DmdAs, sequences showing more than 90% amino acid identity with each other were grouped in the same cluster. Coverage, Simpson’s diversity index and Shannon’s diversity index were generated using the SpadeR program (https://chao.shinyapps.io/SpadeR). Amplicon sequences were annotated using a BLASTX search against NCBI databases (http://www.ncbi.nlm.nih.gov). This analysis was used to distinguish correct target sequences from closely related paralogous sequences and to classify amplicons by cluster. Sequence alignment and phylogenetic tree building were completed using the MEGA 5.05 program with the Poisson model. Neighbour-joining bootstrap tests of phylogeny were run with 1000 replicates.

### Nucleotide sequence accession numbers

The nucleotide sequences obtained in the present study have been deposited in the GenBank database under accession numbers KC864796 (*dddL* gene), KU162982-163012 (*dmdA* genes), KU177047-177209 (*dmdA* genes), KU215540-215580 (*dmdA* genes), KU215581-215594 (*dddP* genes), and KU233485-233520 (*dddP* genes).

## Additional Information

**How to cite this article**: Zeng, Y.-X. *et al.* Diversity of bacterial dimethylsulfoniopropionate degradation genes in surface seawater of Arctic Kongsfjorden. *Sci. Rep.* **6**, 33031; doi: 10.1038/srep33031 (2016).

## Figures and Tables

**Table 1:** Characteristics of water collected from Kongsfjorden in summer 2011.

Station (Location)	Depth (m)	Temperature (°C)	pH	Salinity (psu)	NO_2_ (μmol l^−1^)	NO_3_ (μmol l^−1^)	PO_4_ (μmol l^−1^)	SiO_3_ (μmol l^−1^)	Chlorophyll *a* (μg l^−1^)	Prokaryotes (10^9^ cells l^−1^)
K1 (78°59.286′N, 11°39.459′E)	286	5.3	6.8	33.8	0.12	0.42	0.07	1.44	0.48	1.84
K3 (78°56.973′N, 12°02.557′E)	355	4.5	7.0	34.8	0.12	0.76	0.32	1.56	0.34	1.40
K5 (78°54.176′N, 12°17.750′E)	40	5.1	7.8	30.4	0.21	0.97	0.07	2.09	0.42	3.26

**Table 2:** Number of operational taxonomic units (OTUs) and coverage estimates for clone libraries constructed for seawater samples.

Station	*dmdA* gene clone library					*dddP* gene clone library				
	Number of clones	Number of OTUs	Coverage	Simpson diversity index	Shannon diversity index	Number of clones	Number of OTUs	Coverage	Simpson diversity index	Shannon diversity index
K1	181	82	0.675	0.045	3.79	61	29	0.592	0.251	2.37
K3	32	17	0.631	0.15	2.4	26	14	0.624	0.18	2.21
K5	50	21	0.825	0.08	2.78	ND	ND	ND	ND	ND

ND, no data.

## References

[b1] Curson A. R., Todd J. D., Sullivan M. J. & Johnston A. W. Catabolism of dimethylsulphoniopropionate: microorganisms, enzymes and genes. Nat. Rev. Microbiol. 9, 849–859 (2011). 21986900 10.1038/nrmicro2653

[b2] Sunda W., Kieber D., Kiene R. & Huntsman S. An antioxidant function for DMSP and DMS in marine algae. Nature 418, 317–320 (2002). 12124622 10.1038/nature00851

[b3] Strom S., Wolfe G., Slajer A., Lambert S. & Clough J. Chemical defense in the microplankton II: Inhibition of protist feeding by β-dimethylsulfoniopropionate (DMSP). Limnol. Oceanogr. 48, 230–237 (2003).

[b4] Karsten U., Kirst G. & Wiencke C. Dimethylsulphoniopropionate (DMSP) accumulation in green macioalgae from polar to temperate regions: interactive effects of light versus salinity and light versus temperature. Polar Biol. 12, 603–607 (1992).

[b5] Dickson D. & Kirst G. The role of β-dimethylsulphoniopropionate, glycine betaine and homarine in the osmoacclimation of *Platymonas subcordiformis*. Planta 167, 536–543 (1986). 24240370 10.1007/BF00391230

[b6] Kiene R. P., Linn L. J. & Bruton J. A. New and important roles for DMSP in marine microbial communities. J. Sea Res. 43, 209–224 (2000).

[b7] Kiene R. P., Linn L. J., González J., Moran M. A. & Bruton J. A. Dimethylsulfoniopropionate and methanethiol are important precursors of methionine and protein-sulfur in marine bacterioplankton. Appl. Environ. Microbiol. 65, 4549–4558 (1999). 10508088 10.1128/aem.65.10.4549-4558.1999 PMC91606

[b8] Ledyard K. M., Dacey J. W. & Dacey J. Microbial cycling of DMSP and DMS in coastal and oligotrophic seawater. Limnol. Oceanogr. 41, 33–40 (1996).

[b9] Reisch C. R. *et al.* Novel pathway for assimilation of dimethylsulphoniopropionate widespread in marine bacteria. Nature 473, 208–211 (2011). 21562561 10.1038/nature10078

[b10] Howard E. C. *et al.* Changes in dimethylsulfoniopropionate demethylase gene assemblages in response to an induced phytoplankton bloom. Appl. Environ. Microbiol. 77, 524–531 (2011). 21097583 10.1128/AEM.01457-10 PMC3020546

[b11] Gonzalez J. M., Kiene R. P. & Moran M. A. Transformation of sulfur compounds by an abundant lineage of marine bacteria in the alpha-subclass of the class *Proteobacteria*. Appl. Environ. Microbiol. 65, 3810–3819 (1999). 10473380 10.1128/aem.65.9.3810-3819.1999 PMC99705

[b12] Johnston A. W. *et al.* Molecular diversity of bacterial production of the climate-changing gas, dimethyl sulphide, a molecule that impinges on local and global symbioses. J. Exp. Bot. 59, 1059–1067 (2008). 18281720 10.1093/jxb/erm264

[b13] Todd J. D. *et al.* Structural and regulatory genes required to make the gas dimethyl sulfide in bacteria. Science 315, 666–669 (2007). 17272727 10.1126/science.1135370

[b14] Woodhouse M., Mann G., Carslaw K. & Boucher O. Sensitivity of cloud condensation nuclei to regional changes in dimethyl-sulphide emissions. Atmos. Chem. Phys. 13, 2723–2733 (2013).

[b15] Howard E. C., Sun S., Biers E. J. & Moran M. A. Abundant and diverse bacteria involved in DMSP degradation in marine surface waters. Environ. Microbiol. 10, 2397–2410 (2008). 18510552 10.1111/j.1462-2920.2008.01665.x

[b16] Varaljay V. A., Howard E. C., Sun S. & Moran M. A. Deep sequencing of a dimethylsulfoniopropionate-degrading gene (*dmdA*) by using PCR primer pairs designed on the basis of marine metagenomic data. Appl. Environ. Microbiol. 76, 609–617 (2010). 19948858 10.1128/AEM.01258-09 PMC2805212

[b17] Sun J. *et al.* The abundant marine bacterium *Pelagibacter* simultaneously catabolizes dimethylsulfoniopropionate to the gases dimethyl sulfide and methanethiol. *Nat. Microbiol.* 16065, doi: 10.1038/nmicrobiol.2016.65 (2016). 10.1038/nmicrobiol.2016.65 27573103

[b18] Todd J., Curson A., Dupont C., Nicholson P. & Johnston A. The *dddP* gene, encoding a novel enzyme that converts dimethylsulfoniopropionate into dimethyl sulfide, is widespread in ocean metagenomes and marine bacteria and also occurs in some Ascomycete fungi. Environ. Microbiol. 11, 1376–1385 (2009). 19220400 10.1111/j.1462-2920.2009.01864.x

[b19] Todd J. D. *et al.* DddQ, a novel, cupin-containing, dimethylsulfoniopropionate lyase in marine roseobacters and in uncultured marine bacteria. Environ. Microbiol. 13, 427–438 (2011). 20880330 10.1111/j.1462-2920.2010.02348.x

[b20] Choi D. H. *et al.* Pyrosequencing revealed SAR116 clade as dominant *dddP*-containing bacteria in oligotrophic NW Pacific Ocean. Plos One 10, e0116271 (2015). 25615446 10.1371/journal.pone.0116271 PMC4304780

[b21] Curson A. R., Fowler E. K., Dickens S., Johnston A. W. & Todd J. D. Multiple DMSP lyases in the γ-proteobacterium *Oceanimonas doudoroffii*. Biogeochemistry 110, 109–119 (2012).

[b22] Luce M. *et al.* Distribution and microbial metabolism of dimethylsulfoniopropionate and dimethylsulfide during the 2007 Arctic ice minimum. J. Geophys. Res: Oceans 116 (2011).

[b23] Motard-Côté J. *et al.* Distribution and metabolism of dimethylsulfoniopropionate (DMSP) and phylogenetic affiliation of DMSP‐assimilating bacteria in northern Baffin Bay/Lancaster Sound. J. Geophys. Res: Oceans 117 (2012).

[b24] Svendsen H. *et al.* The physical environment of Kongsfjorden–Krossfjorden, an Arctic fjord system in Svalbard. Polar Res. 21, 133–166 (2002).

[b25] Archer S. *et al.* Contrasting responses of DMS and DMSP to ocean acidification in Arctic waters. Biogeosciences 10, 1893–1908 (2013).

[b26] Cottrell M. T. & Kirchman D. L. Community composition of marine bacterioplankton determined by 16S rRNA gene clone libraries and fluorescence *in situ* hybridization. Appl. Environ. Microbiol. 66, 5116–5122 (2000). 11097877 10.1128/aem.66.12.5116-5122.2000 PMC92431

[b27] Pernthaler A., Preston C. M., Pernthaler J., DeLong E. F. & Amann R. Comparison of fluorescently labeled oligonucleotide and polynucleotide probes for the detection of pelagic marine bacteria and archaea. Appl. Environ. Microbiol. 68, 661–667 (2002). 11823205 10.1128/AEM.68.2.661-667.2002 PMC126737

[b28] De Corte D., Sintes E., Yokokawa T. & Herndl G. J. Comparison between MICRO–CARD–FISH and 16S rRNA gene clone libraries to assess the active versus total bacterial community in the coastal Arctic. Environ. Microbiol. Rep. 5, 272–281 (2013). 23565124 10.1111/1758-2229.12013 PMC3615173

[b29] Howard E. C. *et al.* Bacterial taxa that limit sulfur flux from the ocean. Science 314, 649–652 (2006). 17068264 10.1126/science.1130657

[b30] Iversen K. R. & Seuthe L. Seasonal microbial processes in a high-latitude fjord (Kongsfjorden, Svalbard): I. Heterotrophic bacteria, picoplankton and nanoflagellates. Polar Biol. 34, 731–749 (2011).

[b31] Zeng Y.-X. *et al.* Bacterioplankton community structure in the Arctic waters as revealed by pyrosequencing of 16S rRNA genes. Antonie Van Leeuwenhoek 103, 1309–1319 (2013). 23539199 10.1007/s10482-013-9912-6

[b32] Zubkov M. V. *et al.* Linking the composition of bacterioplankton to rapid turnover of dissolved dimethylsulphoniopropionate in an algal bloom in the North Sea. Environ. Microbiol. 3, 304–311 (2001). 11422317 10.1046/j.1462-2920.2001.00196.x

[b33] Niki T., Kunugi M. & Otsuki A. DMSP-lyase activity in five marine phytoplankton species: its potential importance in DMS production. Mar. Biol. 136, 759–764 (2000).

[b34] Kiene R. P. & Linn L. J. The fate of dissolved dimethylsulfoniopropionate (DMSP) in seawater: tracer studies using ^35^S-DMSP. Geochim. Cosmochim. Ac. 64, 2797–2810 (2000).

[b35] Reisch C. R., Moran M. A. & Whitman W. B. Bacterial catabolism of dimethylsulfoniopropionate (DMSP). Front Microbiol. 2, 172 (2011). 21886640 10.3389/fmicb.2011.00172 PMC3155054

[b36] Yoch D. C. Dimethylsulfoniopropionate: its sources, role in the marine food web, and biological degradation to dimethylsulfide. Appl. Environ. Microbiol. 68, 5804–5815 (2002). 12450799 10.1128/AEM.68.12.5804-5815.2002 PMC134419

[b37] Levasseur M. Impact of Arctic meltdown on the microbial cycling of sulphur. Nature Geosci. 6, 691–700 (2013).

[b38] Kiene R. P. & Bates T. S. Biological removal of dimethyl sulphide from sea water. Nature 345, 702–705 (1990).

[b39] Pinhassi J. *et al.* Dimethylsulfoniopropionate turnover is linked to the composition and dynamics of the bacterioplankton assemblage during a microcosm phytoplankton bloom. Appl. Environ. Microbiol. 71, 7650–7660 (2005). 16332737 10.1128/AEM.71.12.7650-7660.2005 PMC1317407

[b40] Varaljay V. A. *et al.* Bacterial dimethylsulfoniopropionate degradation genes in the oligotrophic north pacific subtropical gyre. Appl. Environ. Microbiol. 78, 2775–2782 (2012). 22327587 10.1128/AEM.07559-11 PMC3318810

[b41] Cui Y. *et al.* Abundance and distribution of dimethylsulfoniopropionate degradation genes and the corresponding bacterial community structure at dimethyl sulfide hot spots in the tropical and subtropical pacific ocean. Appl. Environ. Microbiol. 81, 4184–4194 (2015). 25862229 10.1128/AEM.03873-14 PMC4524131

[b42] Moran M. *et al.* Ecological genomics of marine Roseobacters. Appl. Environ. Microbiol. 73, 4559–4569 (2007). 17526795 10.1128/AEM.02580-06 PMC1932822

[b43] Peng M. *et al.* Phylogenetic diversity of the *dddP* gene for dimethylsulfoniopropionate-dependent dimethyl sulfide synthesis in mangrove soils. Can. J. Microbiol. 58, 523–530 (2012). 22458859 10.1139/w2012-019

[b44] Raina J.-B., Tapiolas D., Willis B. L. & Bourne D. G. Coral-associated bacteria and their role in the biogeochemical cycling of sulfur. Appl. Environ. Microbiol. 75, 3492–3501 (2009). 19346350 10.1128/AEM.02567-08 PMC2687302

[b45] Levine N. M. *et al.* Environmental, biochemical and genetic drivers of DMSP degradation and DMS production in the Sargasso Sea. Environ. Microbiol. 14, 1210–1223 (2012). 22324779 10.1111/j.1462-2920.2012.02700.x

[b46] Simó R., Pedrós-Alió C., Malin G. & Grimalt J. O. Biological turnover of DMS, DMSP and DMSO in contrasting open-sea waters. Mar. Ecol. Prog. Ser. 203, 1–11 (2000).

[b47] Bosshard P. P., Santini Y., Grüter D., Stettler R. & Bachofen R. Bacterial diversity and community composition in the chemocline of the meromictic alpine Lake Cadagno as revealed by 16S rDNA analysis. FEMS Microbiol. Ecol. 31, 173–182 (2000). 10640670 10.1111/j.1574-6941.2000.tb00682.x

[b48] Robarts R. D. & Sephton L. M. The enumeration of aquatic bacteria using DAPI. J. Limnol. Soc. S. Afr. 7, 72–74 (1981).

[b49] Amann R. I., Krumholz L. & Stahl D. A. Fluorescent-oligonucleotide probing of whole cells for determinative, phylogenetic, and environmental studies in microbiology. J. Bacteriol. 172, 762–770 (1990). 1688842 10.1128/jb.172.2.762-770.1990 PMC208504

[b50] Sanders R. W., Porter K. G., Bennett S. J. & DeBiase A. E. Seasonal patterns of bacterivory by flagellates, ciliates, rotifers, and cladocerans in a freshwater planktonic community. Limnol. Oceanogr. 34, 673–687 (1989).

[b51] Comte J. *et al.* Microbial community structure and dynamics in the largest natural French lake (Lake Bourget). Microb. Ecol. 52, 72–89 (2006). 16733620 10.1007/s00248-004-0230-4

